# Observations on spontaneous tumor formation in mice overexpressing mitotic kinesin Kif14

**DOI:** 10.1038/s41598-018-34603-4

**Published:** 2018-11-01

**Authors:** Kamakshi Sishtla, Natalie Pitt, Mehdi Shadmand, Michael N. O’Hare, Rania S. Sulaiman, Anthony L. Sinn, Keith Condon, Karen E. Pollok, George E. Sandusky, Timothy W. Corson

**Affiliations:** 1 Eugene and Marilyn Glick Eye Institute, Indiana University School of Medicine, Indianapolis, IN USA; 2 Department of Ophthalmology, Indiana University School of Medicine, Indianapolis, IN USA; 3 Department of Pathology and Laboratory Medicine, Indiana University School of Medicine, Indianapolis, IN USA; 4 Department of Pharmacology and Toxicology, Indiana University School of Medicine, Indianapolis, IN USA; 5 Department of Pediatrics, Indiana University School of Medicine, Indianapolis, IN USA; 6 Department of Anatomy and Cell Biology, Indiana University School of Medicine, Indianapolis, IN USA; 7 Department of Biochemistry and Molecular Biology, Indiana University School of Medicine, Indianapolis, IN USA; 8 Melvin and Bren Simon Cancer Center, Indiana University School of Medicine, Indianapolis, IN USA; 9 Biomedical Science, University of Ulster, Coleraine, United Kingdom; 10 Department of Biochemistry, Faculty of Pharmacy, Cairo University, Cairo, Egypt

## Abstract

The *KIF14* locus is gained and overexpressed in various malignancies, with prognostic relevance. Its protein product, a mitotic kinesin, accelerates growth of normal mammary epithelial cells *in vitro* and retinoblastoma tumours in a mouse model, while *KIF14* knockdown blocks growth of brain, liver, ovarian, breast, prostate, and other tumour cells and xenografts. However, the tumour-initiating effects of *Kif14* overexpression have not been studied. We aged a cohort of *Kif14*-overexpressing transgenic mice and wild-type littermates and documented survival, cause of death, and tumour burden. The *Kif14* transgene was expressed in all tissues examined, and was associated with increased proliferation marker expression. Neither mouse weights nor overall survival differed between genotypes. However, *Kif14* transgenic mice showed a higher incidence of fatal lymphomas (73 vs. 50%, *p* = 0.03, Fisher’s exact test), primarily follicular and diffuse B-cell lymphomas. Non-tumour findings included a bilateral ballooning degeneration of lens in 12% of *Kif14* transgenic mice but no wild-type mice (*p* = 0.02). Overall, this work reveals a novel association of *Kif14* overexpression with lymphoma but suggests that *Kif14* does not have as prominent a role in initiating cancer in other cell types as it does in accelerating tumour development in response to other oncogenic insults.

## Introduction

The ATP- and microtubule dependent molecular motors known as kinesins have garnered considerable interest as cancer targets. In particular, the mitotic kinesins Eg5 (KIF11) and CENP-E (KIF10) have been inhibited in clinical trials, with some success^1^. However, several other members of the 45-strong kinesin superfamily have been implicated in cancer as well^2^. Prominent amongst these is KIF14, which has an oncogenic role supported by genomic, functional, and clinical evidence^3^. We first identified *KIF14* in a region of somatic genomic gain in retinoblastoma^4^, and subsequently showed low-level amplification of the *KIF14* locus in this cancer^5^, and increasing *KIF14* copy number in the transition from benign retinoma to malignant retinoblastoma^6^. Moreover, the *KIF14* locus is gained in breast carcinoma^5^, hepatocellular carcinoma^5,7^, ovarian carcinoma^8^, papillary renal cell carcinoma^9^, and medulloblastoma^10^. Complementing this genomic gain, *KIF14* is overexpressed in multiple tumour types, including retinoblastoma (both human and murine)^4,11,12^ and cancers of the breast^13^, cervix^14^, liver^15^, lung^16^, ovary^8^, larynx^17^, brain^18,19^, and prostate^20^. Importantly, this overexpression has clinical relevance: high *KIF14* expression is associated with poor prognosis in breast^13^, cervix^14^, liver^15^, lung^16^, ovary^8^, brain^18^, prostate^20^, and other cancers. In breast cancer, *KIF14* expression correlates with proliferation^13^. Consistent with this, knockdown of *KIF14* decreases tumour cell and xenograft growth, while overexpression promotes growth both of cancer cells, and also normal mammary epithelial cells^21^.

The molecular functions of KIF14 are only partially known. It is essential for cytokinesis, and interacts with and helps localize cytokinesis regulators protein regulator of cytokinesis 1 (PRC1) and citron kinase (CIT)^22,23^. In turn, its localization is regulated by Nek7-catalysed phosphorylation^24^. It also may have a cytokinesis-independent role; it can regulate Rap1a-Radil signaling at the cell membrane^25^. At the whole organism level, a spontaneous mouse *Kif14* mutant, *laggard*, revealed that *Kif14* is essential for myelination. *Laggard* mice have microcephaly, markedly reduced brain size, and a severe ataxia phenotype that is lethal within three weeks of birth^26^. Interestingly, *KIF14* loss-of-function mutations in humans have been associated with an embryonic lethal phenotype of microcephaly, renal cystic dysplasia, and genitourinary and brain malformations, traits characteristic of a ciliopathy^27^. More recently, causative *KIF14* mutations have been documented in patients with less severe, non-lethal microcephaly phenotypes^28,29^. This role in microcephaly has been touted as a potential reason to consider KIF14 as a therapeutic target for CNS malignancies^30^.

In characterizing the *laggard* mutant, the Sakisaka group generated a *Kif14* overexpressing transgenic (Tg) mouse, which could rescue the phenotype of *laggard* and *Kif14* knock-out animals^26^. The advent of this model raised the appealing possibility of exploring Kif14’s cancer-promoting effects in animals. We recently crossed this *Kif14* Tg mouse into a transgenic retinoblastoma mouse model. The Simian Virus 40 T-antigen-driven retinoblastoma model is known to upregulate endogenous *Kif14* during tumourigenesis^12^. Transgenic *Kif14* overexpression further accelerated tumour initiation, progression, and total tumour burden in these mice, thus confirming for the first time that *Kif14* promotes tumour growth *in vivo*^31^. We subsequently hypothesized that *Kif14* overexpression might similarly predispose mice to spontaneous tumours. Here, we report the effects of *Kif14* overexpression on tumour formation in a population of otherwise normal mice allowed to live out their lifespans.

## Materials and Methods

### Animals

All animal experiments were approved by the Indiana University School of Medicine Institutional Animal Care and Use Committee and were in accordance with all standards set forth in the Association for Research in Vision and Ophthalmology Statement for the Use of Animals in Ophthalmic and Visual Research. Hemizygous mice constitutively overexpressing *Kif14* (BDF1-Tg(pCAGGS-Kif14)#28; Accession Number CDB0476T, http://www2.clst.riken.jp/arg/TG%20mutant%20mice%20list.html)^26^ or their wild-type BDF1 littermates of both sexes were used for the tumour formation and aging study. Mice were genotyped as described^26^. All pups from consecutive litters entered the study until enrolment numbers were met. In total, 125 mice entered the study (*n* = 61 *Kif14* Tg, 64 wild-type; 2 animals per genotype were subsequently censored due to accidental death). Animals were maintained under standard housing conditions^32^ until natural death or veterinarian-mandated euthanasia by CO_2_ asphyxiation followed by cervical dislocation. Veterinary staff were masked to animal genotype in making euthanasia decisions. Mice were checked daily and were weighed monthly starting at five weeks of age. For gene expression analyses, 5–6-week-old female *Kif14* Tg or BDF1 mice (Charles River Laboratories) were used.

### Necropsy & Histopathology

Necropsies were performed at the Melvin and Bren Simon Cancer Center *In Vivo* Therapeutics Core facility by researchers masked to animal genotype. Tissues collected for histopathology included liver, lung, brain, pituitary, eyes, mammary gland, ovary, seminal vesicle, kidney, spleen, and any abnormal masses. These tissues were fixed in 10% neutral buffered formalin overnight, and transferred to ethanol prior to embedding, sectioning, and H&E staining at the Indiana University School of Medicine Histology Core. Representative sections were read by a board-certified pathologist and two pathology residents masked to genotype. Lymphomas were classified according to the Mouse Models for Human Cancers Consortium “Bethesda” criteria^33^. Histopathological images were taken with an Aperio Slide Scanner.

### Gene expression analysis

We devised a custom qRT-PCR assay to monitor *Kif14* transgene expression without the influence of endogenous *Kif14* expression. The vector used for transgene construction, pCAGGS, contains an intron in the promoter region^34^, so we designed TaqMan primer/probe sequences spanning this intron with the reverse primer within the *Kif14* cDNA sequence to ensure specificity for the transgene without amplification from genomic DNA. Forward primer = 5′-TCTGACTGACCGCGTTACTC-3′, Reverse primer = 5′-CTTCCGA-TGTTGTGTCTGCTATG-3′, and probe = 5′-ACAGCACAATAACCAGCACGTTGC-3′. PCR efficiency for this assay was >90%, and no amplification from *Kif14* Tg or wild-type gDNA, or wild-type cDNA was observed. We used an Applied Biosystems TaqMan assay to monitor endogenous expression of *Kif14* (Mm01291408_m1) in wild-type mice. In order to assess proliferative potential, we utilized a TaqMan assay for *Mki67* (Mm01278617_m1); this gene encodes the proliferation marker Ki-67. Trizol (Invitrogen) was used to isolate RNA from unfixed tissues dissected from healthy *Kif14* Tg and wild-type mice and stored at −80 °C in RNAlater. First-strand cDNA was synthesized from 250 or 500 ng RNA using iScript Select (Bio-Rad) and random primers. PCR reactions included TaqMan Fast Advanced Master mix (Applied Biosystems), and TaqMan primer/probe mixes. For the *Kif14* transgene custom assay, we used 500 nM each primer, and 250 nM probe labeled with FAM and ZEN quencher (IDT). qPCR was performed on a ViiA7 system, and results normalized to housekeeping gene TaqMan assays (Applied Biosystems) for *Tbp* (Mm00446973_m1) and *Hprt* (Mm01545399_m1). Results are reported relative to a pool of all tissues from one mouse.

### Statistical analysis

Mouse survival was compared by the log-rank test. Differential sex ratios and pathologies were assessed by Fisher’s exact test. For *a priori* power calculations, mouse survival was the primary endpoint. Assuming 75% of the transgenic mice and 50% of the non-transgenic mice would develop tumours by 130 weeks and totally 65 mice with tumours (event) would be observed, we had 80% power to detect such difference with *n* = 57 per group at *p* = 0.05 using the log-rank test. Correlation between *Mki67* and *Kif14* expression was assessed by Spearman’s *ρ*. Statistical analyses were performed with GraphPad Prism v. 7.0. Two-sided *p*-values < 0.05 were considered statistically significant in all tests.

## Results

### *Kif14* transgene expression and proliferation

**Figure 1:**
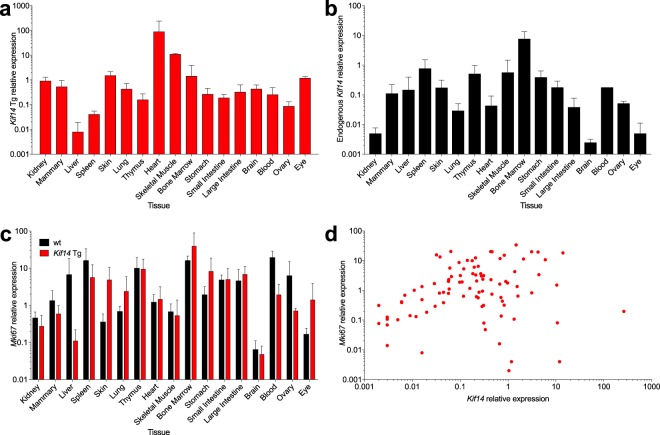
*Kif14* gene expression and proliferation in *Kif14* transgenic (Tg) and wild-type (wt) mice. (**a**) The *Kif14* transgene is expressed in all tissues analyzed in Tg mice. (**b**) Endogenous *Kif14* is expressed in all tissues analyzed in wt mice. (**c**) *Mki67* proliferation marker is increased in some *Kif14* Tg tissues compared with wt tissues. (**d**) *Mki67* expression positively correlates with *Kif14* expression (Spearman’s *ρ* = 0.21, *p* = 0.04). All individual samples from all tissue types analyzed are shown (both wt and Tg mice, endogenous *Kif14* and *Kif14* Tg, respectively). qRT-PCR for the indicated genes, normalized to *Hprt* and *Tbp* and reported relative to a pool of all tissues. Mean ± s.d. of results from 3 individual mice shown. Note differences in y-axis scales between panels.

To assess the *Kif14* transgene expression pattern across tissues, we developed a qRT-PCR assay that would specifically amplify the transgene cDNA. As expected, given the strong “AG” promoter and cytomegalovirus immediate early enhancer used in constructing this strain^26^, we observed *Kif14* transgene expression in all normal tissue types analysed (Fig. 1a). There was considerable variability in expression, however, with highest expression in heart and skeletal muscle, and lowest expression in liver and spleen. In wild-type mice, endogenous *Kif14* was likewise expressed in all tissues examined, with highest expression in bone marrow and spleen, and lowest expression in kidney and brain (Fig. 1b).

Since *Kif14* expression is associated with enhanced proliferation^13^, we assessed expression of the proliferation marker *Mki67* (encoding Ki-67) in all tissues. In some cases, *Mki67* was increased in *Kif14* Tg tissues compared with wild-type counterparts, notably in bone marrow, skin, stomach, lung, and eye (Fig. 1c). Across all tissues, there was a significant positive correlation between *Kif14* levels and *Mki67* levels (Fig. 1d; Spearman’s *ρ* = 0.21, *p* = 0.04).

### Mouse characteristics and survival

**Figure 2:**
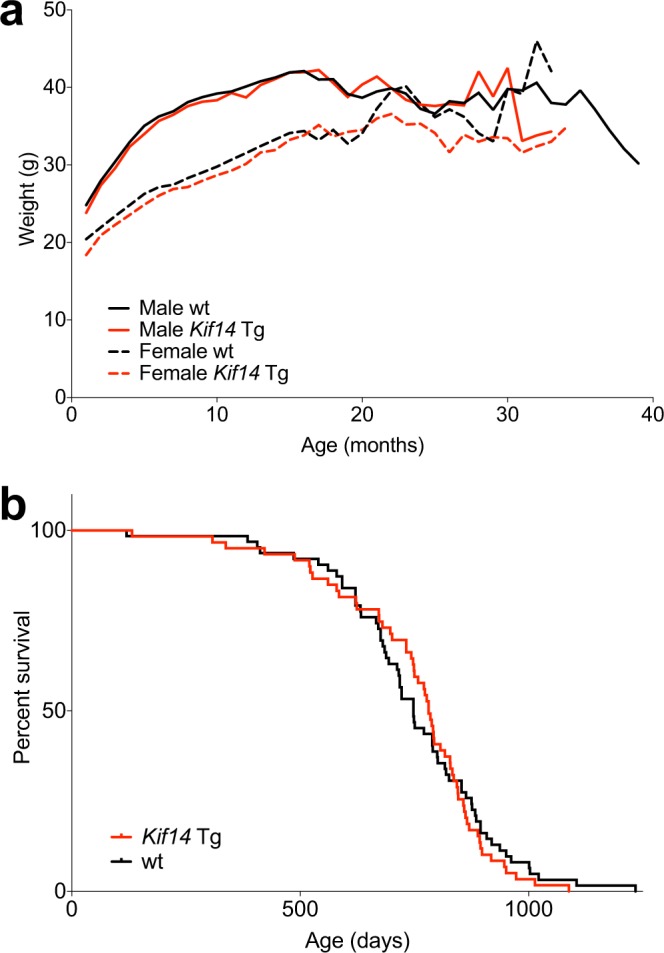
Characteristics of *Kif14* transgenic (Tg) mice. (**a**) Mean weights of *Kif14* Tg mice and their wild-type (wt) littermates, measured monthly through the lifespan. (**b**) Survival of *Kif14* Tg mice and their wt littermates. No survival difference was observed between genotypes (*p* = 0.68, log-rank test).

We monitored the weights and survival of the *Kif14* Tg and wild-type mice to assess *Kif14*’s effects on these parameters. *Kif14* Tg mice were grossly indistinguishable from their wild-type littermates; there was no difference in sex ratio between the two genotypes (*Kif14* Tg: 55% female, wild-type: 45% female; *p* = 0.28) and both genotypes showed an identical pattern of weight gain throughout early life (Fig. 2a). During late life, female *Kif14* Tg mice lost weight somewhat more rapidly than female wild-type mice, but this difference was not observed for their male counterparts. Overall survival also was not influenced by *Kif14* overexpression (Fig. 2b; 781 vs. 748 days median survival for *Kif14* Tg and wild-type mice, respectively, log-rank *p* = 0.68). Age at death ranged from 120–1234 days for wild-type and 308–973 days for *Kif14* Tg mice. Moreover, no survival difference was observed when analysing females and males separately (Females: 777 vs. 722 days median survival, log-rank *p* = 0.64; Males: 788 vs. 749 days median survival, log-rank *p* = 0.92).

### Spectrum of tumours

**Figure 3:**
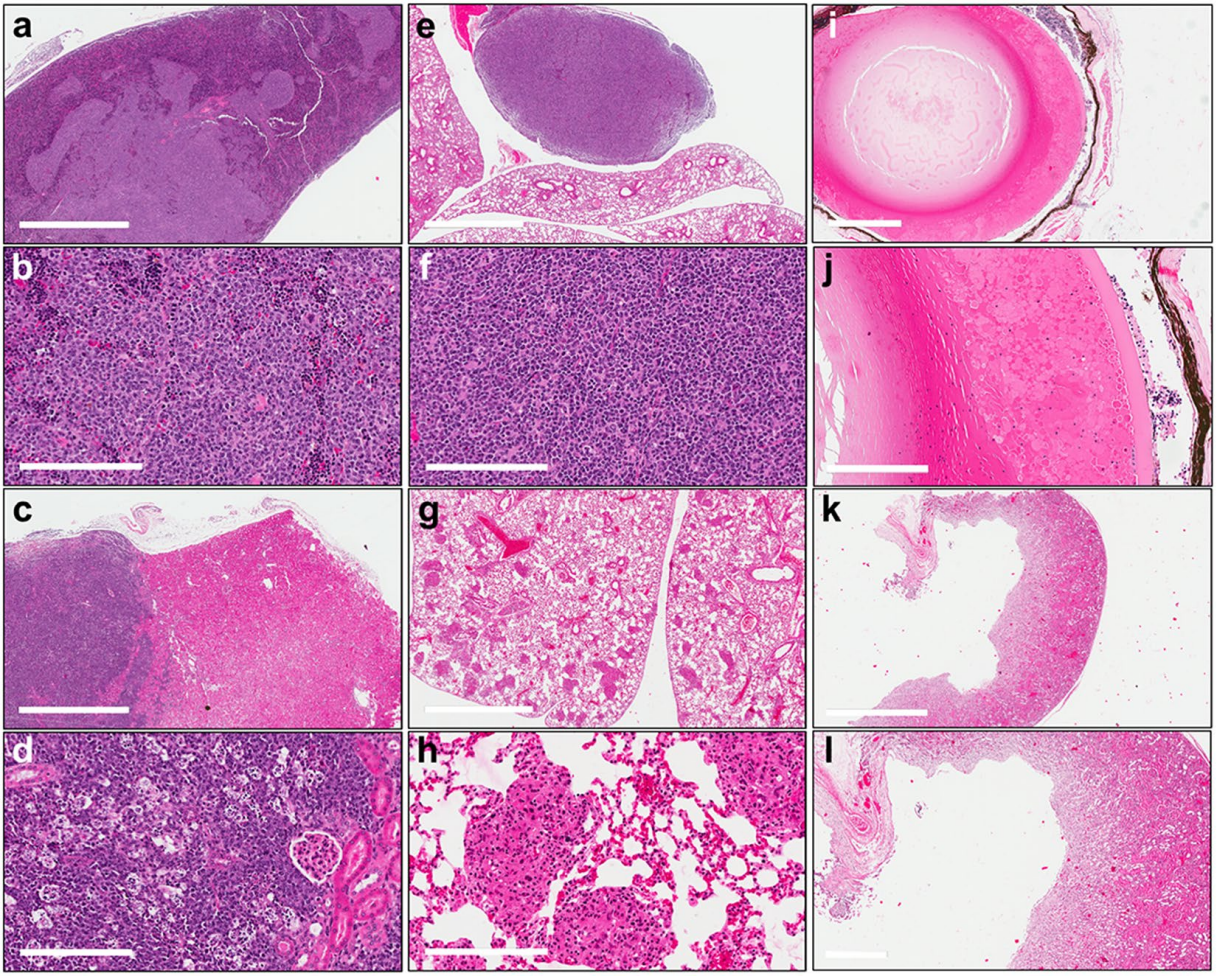
Histology of *Kif14* transgenic mouse tumour and eye phenotypes. Low and high magnification histological H&E images of (**a,b**) follicular lymphoma in spleen; (**c,d**) diffuse large B cell lymphoma in kidney; (**e,f**) thymic lymphoma in lung; (**g,h**) sarcoma in lung; (**i,j**) ballooning degeneration of lens; and (**k,l**) hydronephrosis. Scale bars in (**a,c,e,g,k**) = 2 mm; in (**b,d,f,h,j**) = 200 µm; in (**i,l**) = 600 µm.

Both *Kif14* Tg and wild-type mice developed a wide variety of tumours (Table 1), and there was a non-significantly increased frequency of overall cancer deaths in the *Kif14* Tg group: the presumed cause of death was cancer-related in 90% of the *Kif14* Tg and 78% of wild-type mice for which cause of death could be inferred (*p* = 0.15). Lymphoid malignancies were most common. Follicular lymphomas were the most commonly observed tumours (Fig. 3a,b), followed by diffuse large B cell lymphomas (Fig. 3c,d). Thymic lymphoma was also seen (Fig. 3e,f), as well as varied sarcomas (Fig. 3g,h), carcinomas, myeloid dysplasia, and pituitary tumours. Although total incidence of lymphoma did not differ between genotypes (85 vs. 74%, *p* = 0.29, Table 1), lymphoma as the cause of death was more common in *Kif14* Tg mice than in their wild-type littermates (73 vs. 50%, *p* = 0.03, Table 1). No other tumour type significantly differed between the two groups in incidence or as cause of death (Table 1). Multiple animals bore more than one tumour type at time of death: seven *Kif14* Tg mice had two types of tumours and one *Kif14* Tg mouse had three distinct types of tumours. There were nine wild-type mice with two types of tumours and two wild-type mice with three distinct types of tumours.

**Table 1:** Spectrum of pathological observations in *Kif14* transgenic (Tg) mice versus wild-type (wt) littermates.

Pathology	Observed			Cause of death		
	wt	*Kif14* Tg	*p*	wt	*Kif14* Tg	*p*
**lymphoma**	34 (74)	35 (85)	0.29	23 (50)	30 (73)	***0.03***
follicular lymphoma	14 (30)	19 (46)		10 (22)	16 (39)	
diffuse B cell lymphoma	12 (26)	12 (29)		9 (20)	11 (27)	
thymic lymphoma	5 (11)	3 (7)		4 (9)	3 (7)	
lacrimal gland lymphoma	2 (4)	0 (0)		0 (0)	0 (0)	
unspecified lymphoma	1 (2)	1 (2)		0 (0)	0 (0)	
**sarcoma**	4 (9)	7 (17)	0.34	4 (9)	6 (15)	0.51
undifferentiated sarcoma	1 (2)	2 (5)		1 (2)	1 (2)	
histiocytic sarcoma	1 (2)	3 (7)		1 (2)	3 (7)	
hemangiosarcoma	1 (2)	1 (2)		1 (2)	1 (2)	
fibrosarcoma	1 (2)	0 (0)		1 (2)	0 (0)	
lung sarcoma	0 (0)	1 (2)		0 (0)	1 (2)	
**carcinoma**	8 (17)	2 (5)	0.09	6 (13)	1 (2)	0.11
hepatocellular carcinoma	2 (4)	1 (2)		2 (4)	0 (0)	
adenocarcinoma	4 (9)	0 (0)		2 (4)	0 (0)	
spindle cell carcinoma	1 (2)	0 (0)		1 (2)	0 (0)	
abdominal carcinoma	1 (2)	0 (0)		1 (2)	0 (0)	
ductal carcinoma	0 (0)	1 (2)		0 (0)	1 (2)	
**myeloid dysplasia**	5 (11)	7 (17)	0.54	1 (2)	1 (2)	1.0
**pituitary tumour**	4 (9)	2 (5)	0.41	4 (9)	1 (2)	0.18
**hydronephrosis**	15 (33)	16 (39)	0.65	3 (7)	2 (5)	
**ballooning lens degeneration**	0 (0)	5 (12)	***0.02***	0 (0)	0 (0)	
**hemangioma**	2 (4)	1 (2)	1.0	1 (2)	0 (0)	
**adenoma**	3 (7)	5 (12)	0.47	1 (2)	0 (0)	

Data are stratified into animals in which a pathology was observed, and the subset of animals in which that pathology was the cause of death. Note that some animals exhibited multiple pathologies. Number of animals and (% of total necropsied) per genotype indicated (*N* = 46 for wt, 41 for *Kif14* Tg). Fisher’s exact test *p*-values for comparing genotypes shown; significant differences are bold and italicized.

### Non-tumour pathology

Despite the preponderance of tumours, other non-malignant pathologies were evident in these mice as well. Intriguingly, a bilateral, ballooning degeneration of the lens was seen in five *Kif14* Tg mice but no wild-type mice (*p* = 0.02) (Fig. 3i,j). Other pathologies included hydronephrosis in 30–40% of animals (Fig. 3k,l), adenomas, and hemangiomas (Table 1), plus isolated cases of sepsis and pneumonia. However, the incidence of other pathologies did not differ between genotypes.

## Discussion

*KIF14* is a cancer gene with broad relevance to epithelial and neuronal cancers. Clinical, *in vitro*, xenograft, and transgenic animal studies have indicated that when gained or overexpressed, this gene promotes tumour growth (reviewed in ref.^3^). Beyond the correlative clinical studies, functional examples include accelerated proliferation after overexpression of *KIF14* in ovarian cancer^8^ or hepatocellular carcinoma^35^ cell lines and increased tumor formation in response to *Kif14* overexpression in a mouse model of retinoblastoma^31^. However, *KIF14*’s role as a tumour initiator has not previously been assessed.

Our results suggest that *Kif14* does not act broadly as an initiating oncogene, although may enhance proliferation and severity of cancer, specifically the development of fatal lymphoma, at least in mice. This result is in keeping with findings that *KIF14* overexpression could enhance the proliferation of immortalized human mammary epithelial cells, but did not render them tumourigenic^21^, and with the correlation between *Kif14* expression and *Mki67* expression seen in human breast cancer samples^13^. Here, we found that *Kif14* overexpression moderately enhanced proliferation of cells in the bone marrow, skin, stomach, lung, and eye of *Kif14* Tg mice compared to wild-type mice. Moreover, *Kif14* expression positively correlated with *Mki67* overall. Perhaps in a compromised state (such as after loss of a tumor suppressor gene), the increased proliferation in these tissues is sufficient to enhance the incidence and severity of lymphomas in this mouse model. Thus, *KIF14* appears to be an enabler, accelerating tumour formation and growth against the background of tumour suppressor gene loss and/or other genomic changes^31^. In contrast, overexpression of *Kif11* (Eg5) in mice promoted tumour formation without other oncogenic genetic insults^36^. No overexpressing strains are documented (www.informatics.jax.org) for the other kinesins with known roles in mitosis (KIF2A, KIF2B, KIF2C, KIFC1, KIF4, KIF10, KIF15, KIF18A, KIF18B, KIF19, KIF20A, KIF20B, KIF23, and KIF22)^37^.

Of course, it remains possible that sufficiently high *KIF14* overexpression, beyond the levels attained in this mouse model, or genomic gain/amplification could initiate tumour formation. It will likewise be interesting to assess any reduced incidence of tumours in mice with loss of *Kif14*. Based on the preponderance of evidence from *in vitro* and xenograft studies of *KIF14* knockdown^8,10,15,16,19,20,35^, (partial) loss of *Kif14* would be likely to reduce tumour growth. However, such loss-of-function experiments *in vivo* would require conditional knockouts to bypass the early postnatal lethality seen in constitutive *Kif14* knockouts^26^ and severe CNS phenotypes seen in humans with *KIF14* mutations^27–29^.

The spectrum of neoplasia in the parental BDF1 mouse strain used for creating the *Kif14* Tg was previously described, allowing comparisons with our data^38^. The median survival of mice in our study (107–112 weeks) was similar to that reported (112–118 weeks), although our longest-lived mouse (a wild-type male aged 176 weeks) lived substantially longer than the longest previously reported (158 weeks). Mouse weight curves were also broadly similar. Lymphocytic tumours were most frequent in the previous study (44% of mice), consistent with our findings. However, hepatocellular carcinoma was also prevalent in that study (21% of mice)^38^, but rare in our cohort (4% of wild-type mice). This difference may be due to environmental factors or source of the animals studied. We also did not observe the marked sex differences in tumour incidence previously reported; we observed no difference in tumour frequency between the sexes.

As tumourigenesis was our focus in this study, the non-neoplastic changes we observed were largely incidental. The common hydronephrosis we observed (which did not differ in frequency between genotypes) is perhaps related to the previously-reported renal calculi, proteinaceous casts, and other kidney pathologies seen in BDF1 mice^38^. The lens phenotype, seen exclusively in *Kif14* Tg mice, is more interesting. Since the ballooning degeneration was bilateral, it is likely genotype-related. However, the rarity of this phenotype (~12% of *Kif14* Tg mice) suggests that other genetic or environmental factors beyond *Kif14* overexpression may influence its formation.

Our findings of increased fatal lymphomas in mice overexpressing *Kif14* suggest that further study of this gene in the context of hematopoietic malignancies is warranted. *KIF14* has not been extensively investigated in blood cancers, although both genomic gain of the *KIF14* locus and overexpression have been observed in some human genome-wide lymphoma studies^3^. In particular, one genomic study of human diffuse large B-cell lymphoma showed >30% gain of a genomic region containing *KIF14*^39^, while recent work indicated that the *KIF14* locus was in a genomic region gained in a small cohort of relapsing B-cell precursor acute lymphoblastic leukemia patients^40^. It will be interesting to see if *KIF14* can accelerate growth of human lymphoma cells, or if its loss can block growth of hematopoietic cancers.

In summary, we have shown that although transgenic *Kif14* overexpression does not increase mouse mortality or overall tumor burden, it is associated with an increase in fatal lymphomas, plus a lens phenotype in some cases. *Kif14* overexpression is also associated with proliferation in mouse tissues. Based on these data, *Kif14* perhaps rarely acts as an initiating oncogene. But given its effects on proliferation, and since it has a well-established role in tumour development and progression^3^, it remains an appealing gene for therapeutic targeting in cancer^41^.

## Data Availability

The datasets generated during the current study are available from the corresponding author on reasonable request.

## References

[1] Chandrasekaran G, Tatrai P, Gergely F (2015). Hitting the brakes: targeting microtubule motors in cancer. Br. J. Cancer.

[2] Kozielski, F. (ed.) Kinesins and Cancer. (Springer 2015).

[3] Thériault, B. L. & Corson, T. W. KIF14: a clinically relevant kinesin and potential target for cancer therapy. [F. Kozielski (ed.)] Kinesins and Cancer. 149–170 (Springer 2015).

[4] Corson TW, Huang A, Tsao MS, Gallie BL (2005). *KIF14* is a candidate oncogene in the 1q minimal region of genomic gain in multiple cancers. Oncogene.

[5] Bowles E (2007). Profiling genomic copy number changes in retinoblastoma beyond loss of *RB1*. Genes Chromosomes Cancer.

[6] Dimaras H (2008). Loss of *RB1* induces non-proliferative retinoma: increasing genomic instability correlates with progression to retinoblastoma. Hum. Mol. Genet..

[7] Kim TM (2008). Clinical implication of recurrent copy number alterations in hepatocellular carcinoma and putative oncogenes in recurrent gains on 1q. Int. J. Cancer.

[8] Thériault BL, Pajovic S, Bernardini MQ, Shaw PA, Gallie BL (2012). Kinesin family member 14: An independent prognostic marker and potential therapeutic target for ovarian cancer. Int. J. Cancer.

[9] Szponar A, Zubakov D, Pawlak J, Jauch A, Kovacs G (2009). Three genetic developmental stages of papillary renal cell tumors: duplication of chromosome 1q marks fatal progression. Int. J. Cancer.

[10] Li KK (2017). The kinesin KIF14 is overexpressed in medulloblastoma and downregulation of KIF14 suppressed tumor proliferation and induced apoptosis. Lab. Invest..

[11] Madhavan J (2007). High expression of KIF14 in retinoblastoma: association with older age at diagnosis. Invest. Ophthalmol. Vis. Sci..

[12] Pajovic S (2011). The TAg-RB murine retinoblastoma cell of origin has immunohistochemical features of differentiated Müller glia with progenitor properties. Invest. Ophthalmol. Vis. Sci..

[13] Corson TW, Gallie BL (2006). *KIF14* mRNA expression is a predictor of grade and outcome in breast cancer. Int. J. Cancer.

[14] Wang W., Shi Y., Li J., Cui W., Yang B. (2016). Up-regulation of KIF14 is a predictor of poor survival and a novel prognostic biomarker of chemoresistance to paclitaxel treatment in cervical cancer. Bioscience Reports.

[15] Yang T, Zhang XB, Zheng ZM (2013). Suppression of KIF14 expression inhibits hepatocellular carcinoma progression and predicts favorable outcome. Cancer Sci..

[16] Corson TW (2007). *KIF14* messenger RNA expression is independently prognostic for outcome in lung cancer. Clin. Cancer Res..

[17] Markowski J (2009). Metal-proteinase ADAM12, kinesin 14 and checkpoint suppressor 1 as new molecular markers of laryngeal carcinoma. Eur. Arch. Oto-rhino-laryngol..

[18] Wang Q (2013). Kinesin family member 14 is a candidate prognostic marker for outcome of glioma patients. Cancer Epidemiol..

[19] Huang W (2015). Inhibition of KIF14 suppresses tumor cell growth and promotes apoptosis in human glioblastoma. Cell. Physiol. Biochem..

[20] Zhang Y (2017). Overexpression of a novel candidate oncogene KIF14 correlates with tumor progression and poor prognosis in prostate cancer. Oncotarget.

[21] Singel SM (2014). KIF14 promotes AKT phosphorylation and contributes to chemoresistance in triple-negative breast cancer. Neoplasia.

[22] Carleton M (2006). RNA interference-mediated silencing of mitotic kinesin KIF14 disrupts cell cycle progression and induces cytokinesis failure. Mol. Cell. Biol..

[23] Gruneberg U (2006). KIF14 and citron kinase act together to promote efficient cytokinesis. J. Cell Biol..

[24] Cullati SN, Kabeche L, Kettenbach AN, Gerber SA (2017). A bifurcated signaling cascade of NIMA-related kinases controls distinct kinesins in anaphase. J. Cell Biol..

[25] Ahmed SM (2012). KIF14 negatively regulates Rap1a-Radil signaling during breast cancer progression. J. Cell Biol..

[26] Fujikura K (2013). *Kif14* mutation causes severe brain malformation and hypomyelination. PLoS One.

[27] Filges I (2014). Exome sequencing identifies mutations in *KIF14* as a novel cause of an autosomal recessive lethal fetal ciliopathy phenotype. Clin. Genet..

[28] Moawia A (2017). Mutations of *KIF14* cause primary microcephaly by impairing cytokinesis. Ann. Neurol..

[29] Makrythanasis P (2018). Biallelic variants in *KIF14* cause intellectual disability with microcephaly. Eur. J. Hum. Genet..

[30] Lang PY, Gershon TR (2018). A new way to treat brain tumors: targeting proteins coded by microcephaly genes?. Bioessays.

[31] O’Hare M (2016). *Kif14* overexpression accelerates murine retinoblastoma development. Int. J. Cancer.

[32] Wenzel AA, O’Hare MN, Shadmand M, Corson TW (2015). Optical coherence tomography enables imaging of tumor initiation in the TAg-RB mouse model of retinoblastoma. Mol. Vis..

[33] Morse HC (2002). & Hematopathology subcommittee of the Mouse Models of Human Cancers Consortium. Bethesda proposals for classification of lymphoid neoplasms in mice. Blood.

[34] Niwa H, Yamamura K, Miyazaki J (1991). Efficient selection for high-expression transfectants with a novel eukaryotic vector. Gene.

[35] Xu H (2014). Silencing of KIF14 interferes with cell cycle progression and cytokinesis by blocking thep27(Kip1) ubiquitination pathway in hepatocellular carcinoma. Exp. Mol. Med..

[36] Castillo A, Morse HC, Godfrey VL, Naeem R, Justice MJ (2007). Overexpression of Eg5 causes genomic instability and tumor formation in mice. Cancer Res..

[37] Verhey, K. J., Cochran, J. C. & Walczak, C. E. The kinesin superfamily. *Kinesins and Cancer* [F. Kozielski (ed.)] Kinesins and Cancer. 1-26 (Springer 2015).

[38] Yamate J, Tajima M, Kudow S, Sannai S (1990). Background pathology in BDF1 mice allowed to live out their life-span. Lab. Anim..

[39] Yoon H (2015). Integrated copy number and gene expression profiling analysis of Epstein-Barr virus-positive diffuse large B-cell lymphoma. Genes Chromosomes Cancer.

[40] Ribera J (2017). Copy number profiling of adult relapsed B-cell precursor acute lymphoblastic leukemia reveals potential leukemia progression mechanisms. Genes Chromosomes Cancer.

[41] Basavarajappa HD, Corson TW (2012). KIF14 as an oncogene in retinoblastoma: a target for novel therapeutics?. Future Med. Chem..

